# Evaluating the Proximal Contact Tightness in Direct or In-direct Restoration of Endodontically Treated Teeth: Randomized Clinical Trial

**DOI:** 10.4317/jced.61558

**Published:** 2024-08-01

**Authors:** Sepideh Behzadi, Mahshid Mohammadibassir, Faeze Hamze, Mohammad-Bagher Rezvani

**Affiliations:** 1 Assistant Professor, Department of Operative Dentistry, School of Dentistry, Shahid Beheshti University of Medical Sciences, Tehran, Iran; 2 Associate Professor, Department of Operative Dentistry, School of Dentistry, Shahed University, Tehran, Iran

## Abstract

**Background:**

This study aimed to compare the efficacy of resin composite for establishing a proper proximal contact in comparison to digital work flow Zirconia for restoration of endodontically treated teeth (ETT).

**Material and Methods:**

Forty patients with posterior root canal treated teeth considering the inclusion and exclusion criteria were divided into two groups: half were restored by resin composite while the other received zirconia crown. Then, the proximal contact tightness (PCT) was measured via two methods: 1: VAS: the magnitude of felt PCT was recorded as a number between 0 to 10 (Visual Analogue Scale (VAS)). VAS direct and indirect as VAS-D, and VAS-I respectively. 2: Quantitative: using a custom-made force gage device to record the amount of force needed to pass a mounted dental floss through the proximal contact (Quantitative direct and indirect as Qn-D, and Qn-I respectively) those were compared with the PCT of natural teeth (NT). Data was compared with each other using Chi-square, Shapiro-Wilk, One Way ANOVA, Tukey Post Hoc, Linear regression, and Pearson tests (α= 0.05 in all tests).

**Results:**

There were no significant difference between direct and in-direct groups regarding either sex of the patients (*P*= 0.10), type of teeth (*P*= 0.32), or jaw side (*P*= 0.36). The VAS-D and VAS-I showed similar results in pairwise comparison (*P*= 0.21). Moreover, both the Qn-D and Qn-I showed significantly higher PCT comparing to NT (*P*= 0.45 and 0.0.0001 respectively) while the Qn-D and Qn-I were not distinguishable statistically (*P*= 0.23). Furthermore, significant correlation was observed between VAS and quantitative methods for evaluation of PCT (Pearson *P* value= 0.005).

**Conclusions:**

Both the direct and in-direct restorations lead to clinically acceptable PCT, whilst indirect restorations showed slightly better results which was not statistically noticeable.

** Key words:**Composite resins, Zirconium oxide, Digital Technology.

## Introduction

Dentists always face a challenge to restore the endodontically treated teeth (ETT) because in these situations most of the tooth structure has been lost due to caries progression, or traumatic injury beside access preparation for root canal treatment (RCT) (1). Actually, restorative complication has been documented as the most common reason for extraction of ETT (2,3). Therefore, seeking for the best restorative option is always a subject of interest in dentistry investigations.

Decision making to choose the appropriate restoration for ETT depends on the residual tooth structure particularly in peri-cervical region; which is historically defined as ferrule effect (4-6). Although some studies suggest in-direct restorations for better survival of ETT (7), many others believed direct restorations are also accepTable (8,9). Various literatures report clinical success rate for either direct or indirect restorations in ETT (10-12). Besides that, in modern dentistry, conservative approach for both RCT (13,14) and restoration of ETT has been dramatically emphasized in order to holistically preserve the most tissue as possible (15). Moreover, it has been shown that most retrospective clinical studies, which suggest the in-direct restorations of ETT in their results, suffer from an important bias; they chose indirect restorations for samples with more favorable prognosis while direct filling were done in teeth with questionable prognosis (15). Nevertheless, cuspal coverage should be definitely considered in both restorative models (15).

The direct restorations have numerous advantages including: one session appointment, low cost, easy to apply, and conservation of tooth structure (16). Meanwhile, during reconstructing the crown of an ETT, establishing a suiTable occlusal anatomy, proximal contour and contact with neighboring teeth, maintaining the periodontal health, optimal esthetic, and preserving the tooth structure from future catastrophic fracture are crucial factors (17).

On the other hand, many literatures state that managing a perfect direct restoration on posterior ETT makes trouble for clinician in most cases because reconstruction of optimal proximal contact and contour is quite difficult in extensive cavities even with incorporation of advanced wedge and matrix systems (18). Conversely, others argued that proper proximal contact could be established also by direct restorations (19).

Since open contact leads to food impaction, gingival problem, and tooth movement, tight proximal contact is crucial in permanent dentition (20,21). Various investigations have been conducted seeking for best material and equipment to create a proper proximal contact (19-22). Since ETT have often lost one or two of their proximal surfaces, reconstructing the proximal contact would be an important criterion in either direct or indirect restorative approaches.

Therefore, the aim of this study consists of comparing direct resin composite and indirect digital work flow ceramic to restore the proximal contact tightness (PCT) in posterior ETT via quantitative and Visual Analogue Scale (VAS) methods. Our null hypothesis consists.

1: The PCT is similar in direct and indirect groups.

2: The qualitative and VAS methods are not compatible with each other.

## Material and Methods

The study was approved by local Ethics Committee (IR.SHAHED.REC.1401.171 (Shahed University, Tehran, Iran)) and was registered at the “Iranian Registry of Clinical Trials virtual platform” (IRCT) as IRCT20230311057684N1.

-Sample size calculation

**Figure 1:**
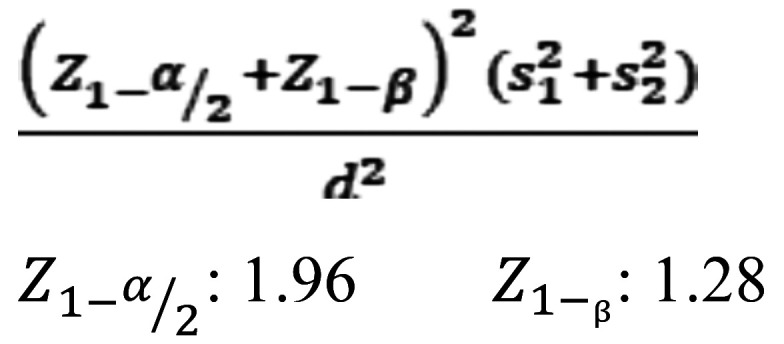
Formula.

According to Wirsching *et al*. (23), the following formula was used while the level of significance considered as 0.05, and the power as 90% (Fig. 1):

m1 ± s1 = Mean differences in proximal contact strengths ± standard error of the mean of group 1(Separation ring (Palodent))= 0.98±1.07

m2± s2 = Mean differences in proximal contact strengths ± standard error of the mean of group 2: (Wedge and Tofflemire retainer) = -0.25±1.00

d=m1-m2

The sample size was obtained as 15 in the formula. But, considering the possible dropouts, 20 restorations of each technique were performed.

-Custom made force meter device and pilot study

**Figure 2:**
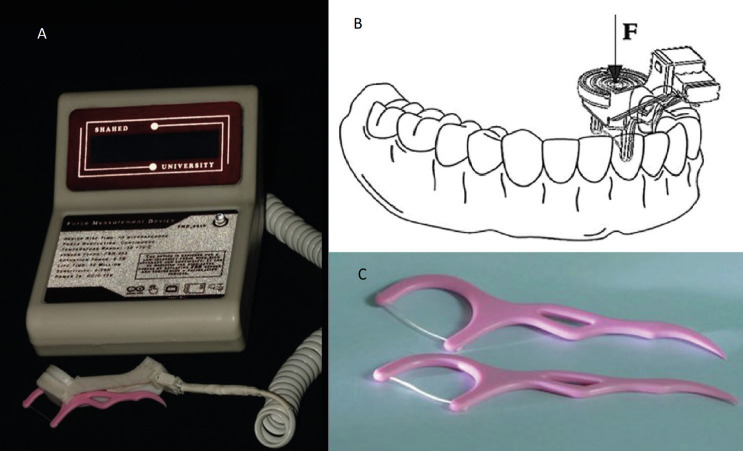
A) Fully assembled costume made digital force meter. B) schematic picture of incorporating the device in the oral cavity. C) Dental floss with floss holder.

**Figure 3:**
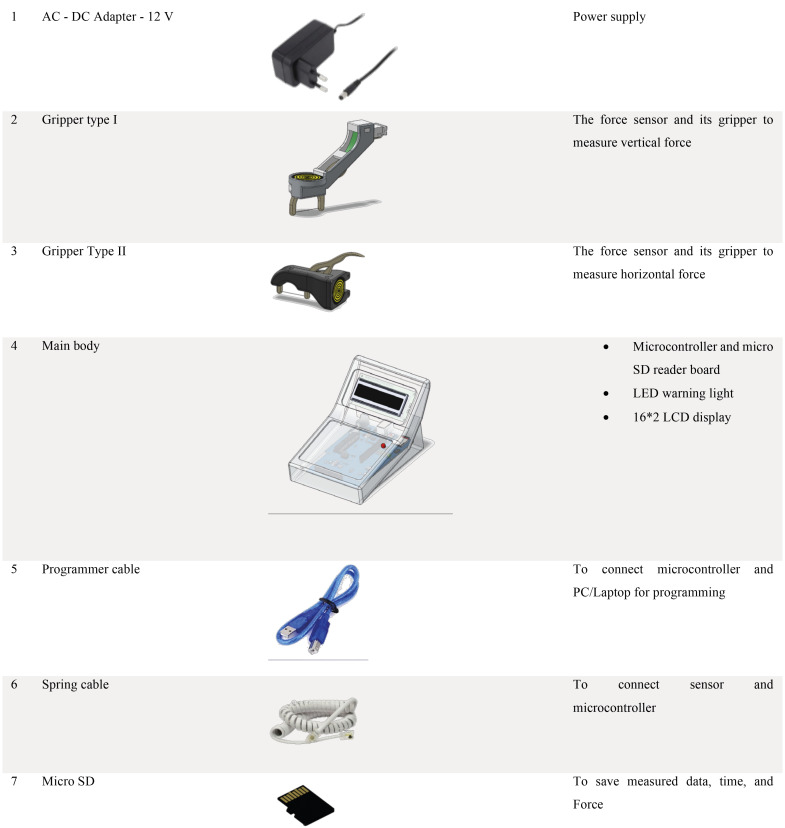
Components of the costume made digital force meter.

A custom digital force meter containing a dental floss was made as described in Fig. 2A and its details are represented in Fig. 3.

At the beginning, the accuracy of the device was evaluated in a pilot study. In which 20 patients were selected who had at least one proper proximal contact between the natural tooth. The PCT of the natural teeth were recorded by the made device and triple repeated (considered as quantitative) (Fig. 2B). Simultaneously, another operator (Operative dentist with 15 years of experience) were asked to pass a similar dental floss (Ever clean dental floss picks, Iran) (Fig. 2C) through the proximal contact and record the felt tightness as a number between 0 to 10 (considered as VAS). Finally, the correlation between the obtained data from these two methods was evaluated.

-Patient selection

All the patients who priorly received posterior teeth RCT in endodontic department in the last two weeks were referred to operative dentistry department (Shahed Dental School, Shahed University, Tehran, Iran). Among them, the patients were selected according to the following inclusion and exclusion criteria.

Inclusion criteria:

The patients should age between 12-70 years old, permanent dentition, good oral health, and normal occlusion (19,24-26).

At least one posterior tooth received proper RCT (no clinical failure, nor apical radiolucency) (27).

At least one proximal surface (mesial or distal) of ETT should be lost due to caries progression or old restoration (an entirely open proximal contact) (9).

The adjacent tooth should exist to form the proximal contact (19).

The ETT should have at least two remaining vertical walls with more than 3 mm thickness (15).

The patient should sign the informed consent form (24).

Exclusion criteria.

Severe systemic or mental disorder, allergy, pregnancy or lactation, any soft, or hard tissue lesion in oral cavity (26).

Severe mal-occlusion (short span dental arch, or generalized diastema) (25).

Bruxism or parafunction (28).

TMJ disorders (19).

Severe periodontic problem (periodontal attachment loss ≥ 40%) (25).

Un-controllable bleeding on probing (Gingival index score more than 1) (25).

Pathologic mobility (grade ≥ 2) of the ETT, or its adjacent tooth (26).

-Randomization:

The blocked randomization approach was used in this study. We asked a person who was not involved in the study to write 20 odd and 20 even numbers on separate cards and embed them in opaque envelopes. At the beginning of the treatment, one pocket was opened by the clinician; the odd numbers dictated direct restoration while the even dictated indirect. Each pocket was withdrawn after usage and it had no substitute (19).

-Direct restoration:

After removing the temporary restoration, and possible remaining caries, each tooth cusp adjacent to the lost marginal ridge was reduced 1.5mm if it was non-functional, or 2mm if functional (in the case that both marginal ridges were lost, all the cusps were reduced). The circumferential metal matrix (Temrex, 0.0015 inch, JR Rand Dental, NY, USA) was burnished to produce an occluso-gingival convexity, mounted in Tofflemire holder, and placed while it was supported by a suitable size of anatomic wooden wedge (Anatomical Dental Wedge, Mina, Iran). Thereafter, the enamel margins were etched selectively for 5 s (Ultraetch 35%, Ultradent, U.S.A), washed for 5 s, bonding agent was applied according to the instruction (Ambar Universal APS, FGM, Joinville, SC, Brazil), and light cured for 20 s (BLUEDENT Smart, BG Light Ltd, Plovdiv, Bulgaria). Subsequently, multiple layers of resin composite (Vittra APS, FGM, Joinville, SC, Brazil) was inserted in the cavity (2mm thickness while the most gingival layer was 1mm) and adapted to the cavity wall. The clinician pushed the matrix band toward the adjacent teeth using a proper hand instrument (Contact Pro contact forming instrument, CEJ Dental Inc, USA) prior to and also during the application of the light cure for 40 s on each layer. Moreover, the additional 40s light curing was applied on each buccal and lingual surface after removing the wedge and matrix band. Finally, occlusal adjustment, finishing, and polishing of the restoration was accomplished by burs and rubbers respectively (red and yellow band Taper, needle, and football shape diamond burs in order (Drendel+Zweiling Diamant GmbH, Kalletal, Germany)) (Pink, green, and white rubber points and cups in order (Kenda polishers, Coltene Whaledent, USA)).

**Figure 4:**
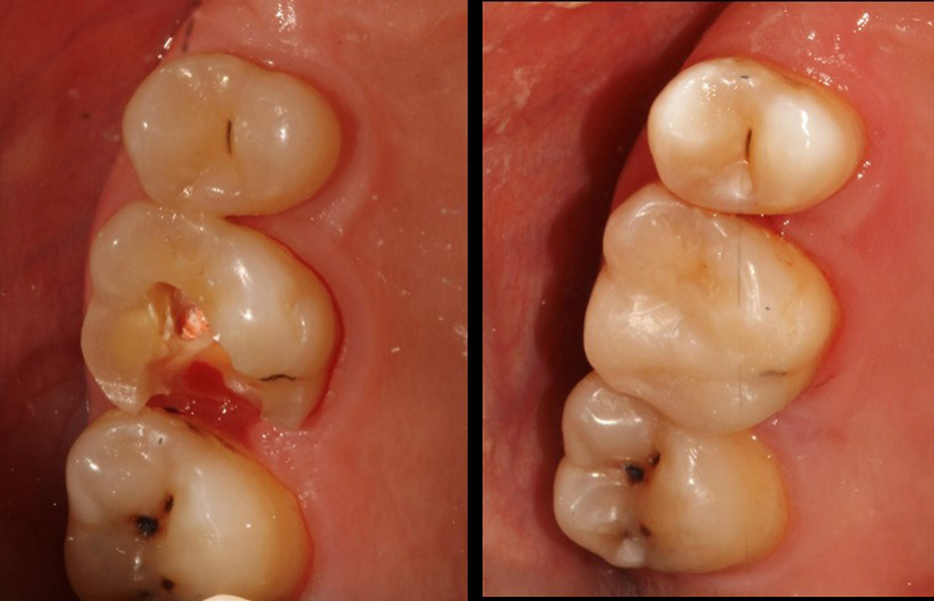
Direct restoration on upper right first molar (before and after composite buildup).

A sample photograph of before and after of the direct restoration is represented in Fig. 4.

-Indirect restoration.

After removing the temporary restoration, and possible remaining caries, the largest canal of the tooth (Distal canal in mandibular molars, Palatal in maxillary molars and premolars) was prepared, the best fitted fiber post (Glassix, Harald Nordin sa, Chailly/Montreux, Switzerland) was cemented (Panavia F 2.0, Kuraray Noritake, Okayama, Japan), and core build up was accomplished by resin composite (Vittra APS, FGM, Joinville, SC, Brazil). Thereafter, the axial and occlusal surfaces were reduced 0.8 and 1.5 mm respectively using round end coarse taper bur (Drendel+Zweiling Diamant GmbH, Kalletal, Germany) mounted in high speed handpiece (NSK, Japan). Retraction cord was placed circumferentially in gingival sulcus, and the prepared tooth (including the whole related quadrant), its antagonist quadrant, and two jaws in occlusion were scanned optically by an oral scanner (iAton, 88dent, Pero, Italy). Subsequently, a temporary restoration was adjusted on the prepared tooth (Acropars TR2, Marlic Medical Inc., Eshtehard, Iran).

After importing the PLY file into the Exocad 3.1 Rijeka software (Exocad GmBH, Darmstadt, Germany), the anatomic crown was designed by the same clinician and the final file was emailed to dental lab to mill the monolithic Zirconia (XangTech 3D Pro Multilayer Zirconia Block, Nanyang Liandong Biotechnology Co., China).

Finally, the prepared restoration was cemented using a dual cured self-etch resin cement (Panavia F 2.0, Kuraray Noritake, Okayama, Japan) according to the manufacturer’s instruction.

**Figure 5:**
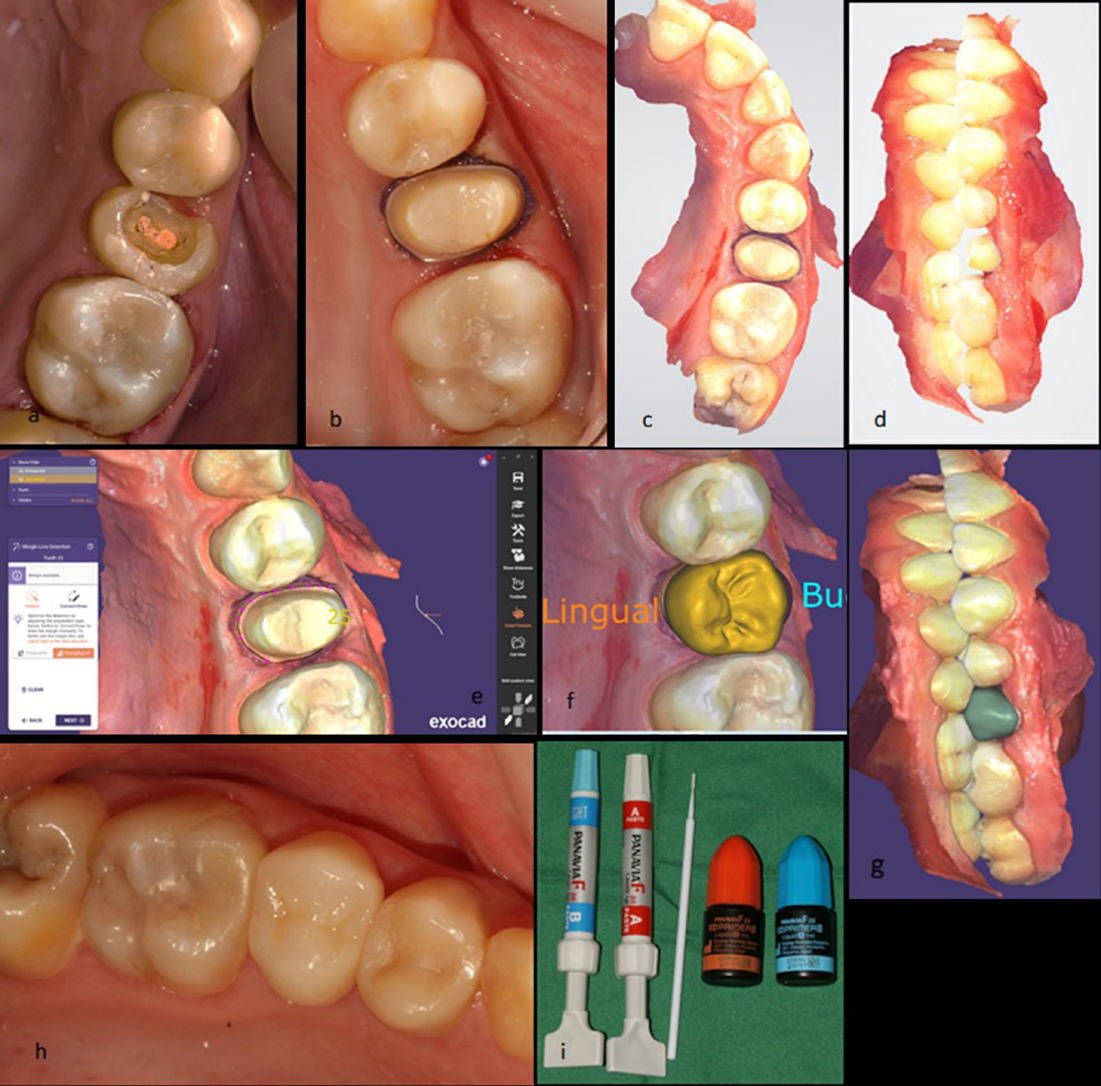
Indirect restoration fully digital workflow: Upper left second premolar was prepared for receiving monolithic Zirconia full coverage crown (a,b). Scanning was done using Intra-oral scanner (c,d). Restoration design was done in Exocad software (e,f,g). After try-in, the restoration was cemented with Panavia F 2.0 (h,i).

The process of a sample patient is depicted in Fig. 5.

-Visual Analogue Scale (VAS) measurement of the proximal contact:

The patient was seated on dental unit positioned at 45° angle and rest his/her head. An operative dentist (clinical experience since 2008 till now) who was calibrated before the study, passed an available commercial dental floss mounted on a plastic holder (Ever clean dental floss picks, Iran) (Fig. 2C) through the proximal contact and recorded the felt tightness as a number between 0 to 10 in VAS (0 representing no contact at all, and 10 as the tightest). Each floss was used for just one restoration. This method served as VAS measurement in two sub groups: VAS-D (VAS-direct), and VAS-I (VAS-Indirect).

-Quantitative recording of the proximal contact.

The patient positioned as mentioned above and similar floss was mounted in the force meter device. Each floss was disposed after assessing one restoration.

The same clinician passed the floss through the proximal contact while another clinician who was blinded about the VAS data, record the amount of force demonstrated on the device monitor. The whole procedure was repeated three times in each contact point and the average force was recorded (N).

This method served as quantitative measurement in two sub groups: Qn-D (Quantitative-direct), and Qn-I (Quantitative-Indirect) which were compared to natural tooth group (NT) (data obtained from the above-mentioned pilot test).

-Statistical analysis:

Comparison of the descriptive data (distribution frequency of patient and studied tooth) were accomplished via Chi-square test.

After evaluating the normal distribution of data using Shapiro-Wilk test. In case of normal distribution, the quantitaive data were compared using One Way ANOVA and Tukey Post Hoc tests. Meanwhile the VAS data in two groups (VAS-D, and VAS-I) were compared with each other after considering a cut-off point (7th scale of VAS) using Chi-square test.

Moreover, the linear regression was done in order to evaluate the effect of different descriptive variables (Sex, type of studied tooth, and jaw side) on obtained PCT.

Also, Pearson test was incorporated for assessing the correlation between two approaches of recording the PCT (VAS and quantitative).

In all mentioned tests, the significance level was set as 0.05 (α= 0.05).

## Results

**Figure 6:**
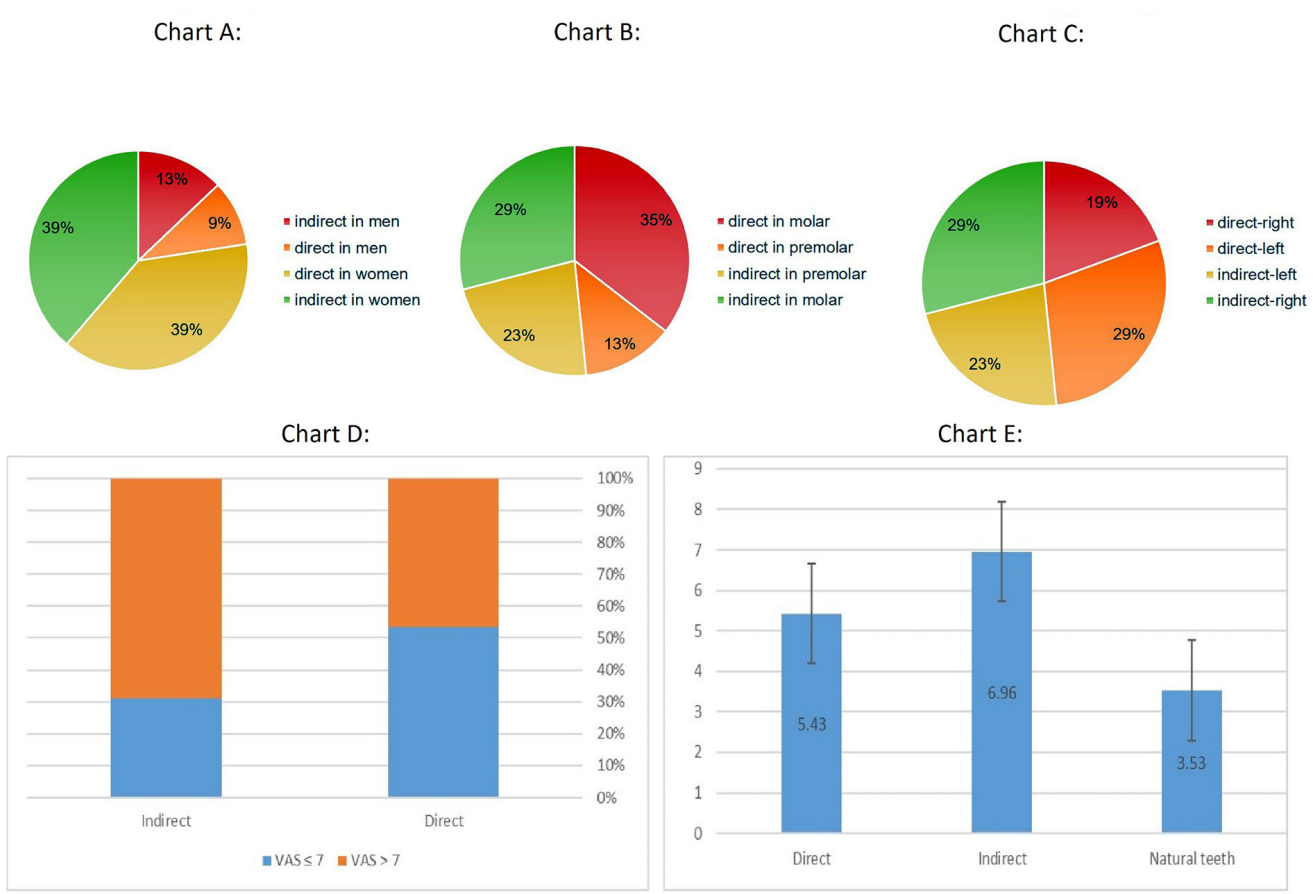
Distribution of patients in Direct and In-direct groups according to gender (Chart A), tooth type (Chart B), and jaw side (Chart C). Chart D: Visual Analogue scale (VAS) assessment of the proximal contact tightness (PCT) in two groups showing the percentage of specimens above or beneath the cut of point (the 7th scale was considered as cut of point). Chart E: Mean ± S.D of Quantitative analysis of the proximal contact tightness (PCT) in direct (Qn-D), indirect (Qn-I), and natural teeth (NT) sub-groups.

-Descriptive data:

After elimination of un-qualified data and withdrawing the un-cooperative patients, the data related to 31 restorations (15 direct and 16 indirect) was compared with each other. Among which, 7 (25.9%) were men while 20 (74.1%) were women; 20 (64.5%) teeth were molar while 11 (35.5%) were Pre-molar; and 15 (48.4%) teeth were located on the right side of the jaw while 16 (51.6%) were on the left. The pie charts related to these distributions are presented in Fig. 6.

The chi-square test revealed no significant difference between direct and in-direct groups regarding either sex of the patients (*P*= 0.10), type of teeth (*P*= 0.32), or jaw side (*P*= 0.36).

-Comparing the PCT in different sub-groups:

Our statistical analysis revealed normal distribution in all sub-groups except VAS-I (*P*= 0.21, 0.13, 0.71, and 0.003 for Qn-D, Qn-I, VAS-D, and VAS-I respectively). However, since the VAS data were ordinal, the non-parametric test was chosen for comparing them.

Fig. 6 demonstrate the distribution of case in VAS-D and VAS-I sub-groups when the 7th scale of VAS was considered as the desired cut-off point (VAS values of 7 or higher were regarded as proper clinical PCT), in VAS-D subgroup, 46.7% and in VAS-I 68.8% of restorations had 7 or higher VAS values. The Chi-square test revealed no significant difference among these two sub-groups (*P*= 0.21). In fact, both the VAS-D and VAS-I were able to reproduce clinically acceptable PCT.

On the other hand, Fig. 6 demonstrate the mean ± S.D of Qn-D, Qn-I, and NT sub-groups. As can be seen, both the Qn-D and Qn-I had higher PCT comparing to NT. Precisely, One Way ANOVA revealed significant difference between three groups (*P*= 0.000), and Tukey Post Hoc showed that both Qn-D and Qn-I were statistically distinguishable from NT (*P*= 0.045 and 0.0.0001 respectively) while the Qn-D and Qn-I did not have significant difference with each other (*P*= 0.23).

-Effect of different variables on PCT in different sub-groups:

The linear regression revealed that none of sex, type of tooth, and jaw side variables had significant effect on PCT in either sub-groups except in VAS-I; in which just the jaw side showed significant effect on the result (*P*= 0.27, 0.67, 0.77 for Qn-D, *P*= 0.84, 0.62, 0.26 for Qn-I, *P*= 0.20, 0.17, 0.53 for VAS-D, and *P*= 0.43, 0.18, 0.03 for VAS-I sub-groups regarding sex, type of tooth, and jaw side variables respectively (ß = 2.202 for the latter P)).

-Correlation between two methods.

There was significant correlation between VAS and quantitative methods for evaluation of PCT (Pearson *P* value= 0.005). Therefore, these two methods including the VAS scale and custom-made digital force meter (quantitative) are compatible with each other. Hence, our innovative device was as reliable as the VAS scale.

## Discussion

Our results revealed that there was no statistically significant difference between the PCT in direct or in-direct groups.

This finding is in agreement with many previous documentations which reported similar clinical performance for both direct resin composite and indirect restorations (ceramic, metal, or porcelain fused to metal) (15,29,30). However, none of these literatures focused on proximal contact. But they investigated many parameters among which the proximal contact was also included and by analyses of all parameters they reported similar clinical success rate for both direct and indirect restorations. Nevertheless, in a clinical investigation, Torres *et al*. compared two approach for resin composite restorations (one group was completely direct placement while the other consist of semidirect composite restoration); and found similar results for the PCT in both groups at seven days after placement (19). Their findings were similar to our findings.

Moreover, our study showed that incorporating the simple circumferential matrix and Tofflemire holder system could reconstruct a proper PCT in direct composite restorations, which was interestingly comparable to digital work flow indirect restorations. Actually, we used the mentioned matrix since it is the most common system in most dental offices; one clinical study reported that the circumferential matrix was preferred by 83% of dentists (20). Hence, our outcome about the matrix could be considered quite beneficial clinically in which the most traditional and simplest system (circumferential matrix and Tofflemire holder) showed similar results compared to the most modern technique (digital work flow).

We incorporated the quantitative method as well as VAS in order to measure the PCT, while most of the studies used only the qualitative method (15,19,20,29,30). In qualitative methods, the PCT was graded by an expert clinician (15,19,29,30). As mentioned above, we used the VAS for measuring the PCT and VAS scales ≥ 7 were considered as clinically accepTable. We chose the 7th scale as the cut-off point because we considered it as an appropriately tight contact. Essentially, in order to be on the safe side and to be strict in our labeling as clinically accepTable, we chose this cut-off point. However, our results showed that even with this strict point of view, the direct and indirect restorations could lead to similar PCT.

Meanwhile, in some studies, the contact point was just classified as open, or closed (20) that could not be enough for clinical judgment because the loose proximal contact would be as harmful as an open contact (20,21).

The quantitative method was also included in some previous literatures (21,26,31,32). Some of them were laboratory studies in which the universal testing machine (Instron machine) was incorporated to report the exact value of applied force for measuring the PCT (21,31). This method is obviously not applicable in clinical situations. Besides that, there are also clinical researches in which the PCT was measured via a force meter (26,32). However, they applied a lateral force (directed from lingual to buccal) to pass a strip between the teeth for measuring the PCT. This manner of using the device, as they presented in their pictures, the measurement would face a big bias in the posterior region because the device would get caught in the patient’s cheek during the lateral buccolingual movement (26,32) and the reported maximum frictional force could face a bias. But we used the vertical force (occluso-gingival direction) for measuring the PCT and we omitted the mentioned possible bias.

It should be emphasized that our quantitative method was achieved by a custom-made device in which the dental floss was mounted. Some studies state that the dental floss is not good for measuring the proximal contact tightness because its diameter is completely dependent on the operator’s applied force, but it is the most common technique (22). Actually, one study showed that most dentists only know this method for evaluation of the proximal contact and they are not familiar with the other methods (22). Hansen *et al*. recorded that almost all of the dentists (34 of 35 dentists) enrolling in their study, used dental floss for assessing the proximal contact (22). In more detail, we used the dental floss for both the VAS and quantitative tests. But, for compensating the amount of applied force and the for stretching of the dental floss, we used the available commercial floss mounted on to a plastic holder (Fig. 4C); therefore, exactly the same length of floss was incorporated in all samples while just vertical force was applied to pass it through the contact point (no force was applied to stretch the floss parallel to its longitudinal axis). It should be highlighted that the clinician was asked to hold the floss perpendicular to the tooth’s long axis.

Furthermore, one of the most noteworthy points in our study is the fact includes that, the indirect restoration was designed by the same clinician who performed the direct restoration. Therefore, the role of the laboratory technician was lowered to the least amount possible.

Conversely, the most important limitation of our study was the fact that we assessed the contact point right after removing the matrix band and we did not follow the cases after several months. So, we strongly suggest clinical follow up for evaluating the durability of established proximal contact in future studies.

## Conclusions

Under the limitations of the current study, our randomized clinical trial showed that an acceptable proximal contact could be established either by direct or in-direct restorations. Although indirect restorations showed slightly better results, the difference was not statistically significant.

## Data Availability

The datasets used and/or analyzed during the current study are available from the corresponding author.
